# Trilobatin Alleviates Cognitive Deficits and Pathologies in an Alzheimer's Disease Mouse Model

**DOI:** 10.1155/2021/3298400

**Published:** 2021-11-05

**Authors:** Jiuyang Ding, Jian Huang, Dan Yin, Ting Liu, Zheng Ren, Shanshan Hu, Yuanliang Ye, Cuiyun Le, Na Zhao, Hongmei Zhou, Zhu Li, Xiaolan Qi, Jiang Huang

**Affiliations:** ^1^School of Forensic Medicine, Guizhou Medical University, Guiyang 550004, China; ^2^School of Forensic Medicine, Southern Medical University, Guangzhou 510515, China; ^3^Laboratory of Electron Microscopy, School of Basic Medicine, Guizhou Medical University, Guiyang 550004, China; ^4^State Key Laboratory of Functions and Applications of Medicinal Plants, Key Laboratory of Pharmaceutics of Guizhou Province, Guizhou Medical University, Guiyang 550004, China; ^5^Good Clinical Practice Center, Affiliated Hospital of Zunyi Medical University, Zunyi 563003, China; ^6^Department of Neurosurgery, Liuzhou People's Hospital, Liuzhou, China; ^7^Key Laboratory of Endemic and Ethnic Diseases, Ministry of Education, Guizhou Medical University, Guiyang 550004, China

## Abstract

Alzheimer's disease (AD) is the most common neurodegenerative disease nowadays that causes memory impairments. It is characterized by extracellular aggregates of amyloid-beta (A*β*), intracellular aggregates of hyperphosphorylated Tau (p-Tau), and other pathological features. Trilobatin (TLB), a natural flavonoid compound isolated from *Lithocarpuspolystachyus* Rehd., has emerged as a neuroprotective agent. However, the effects and mechanisms of TLB on Alzheimer's disease (AD) remain unclear. In this research, different doses of TLB were orally introduced to 3×FAD AD model mice. The pathology, memory performance, and Toll-like receptor 4- (TLR4-) dependent inflammatory pathway protein level were assessed. Here, we show that TLB oral treatment protected 3×FAD AD model mice against the A*β* burden, neuroinflammation, Tau hyperphosphorylation, synaptic degeneration, hippocampal neuronal loss, and memory impairment. The TLR4, a pattern recognition immune receptor, has been implicated in neurodegenerative disease-related neuroinflammation. We found that TLB suppressed glial activation by inhibiting the TLR4-MYD88-NF*κ*B pathway, which leads to the inflammatory factor TNF-*α*, IL-1*β*, and IL-6 reduction. Our study shows that TLR4 might be a key target of TLB in AD treatment and suggests a multifaceted target of TLB in halting AD. Taken together, our findings suggest a potential therapeutic effect of TLB in AD treatment.

## 1. Introduction

Alzheimer's disease (AD) is the most common neurodegenerative disease in the elderly [1]. The AD patient number will reach 100 million worldwide by 2050, which creates an enormous burden on societies and families [2]. Evidence shows that amyloid-*β* (A*β*), oxidative stress, synaptic degeneration, and neuroinflammation play central roles in AD progression [3, 4]. However, drugs that target A*β* deposition or Tau phosphorylation would not efficiently halt AD progression in recent years [5, 6]. Besides synthetic drugs, many traditional Chinese medicines and natural products are found available for AD management [7–12]. These research studies shed new light on the discovery of natural products to alleviate AD pathologies.

Recently, there has been increasing evidence showing that the immune system function alters in AD cases [13]. In AD patients, chronic neuroinflammation is remarkable and leads to immune system dysfunction [14]. Toll-like receptors (TLRs) are a class of highly conserved receptors that modulate innate immune responses [15]. Among the 13 TLRs in mammals, the Toll-like receptor 4 (TLR4) has been well studied in neurodegenerative disease. TLR4 can activate downstream signals through MYD88 and non-MYD88 pathways [16]. The nuclear factor kappa B (NF*κ*B) promoter, which transcribes inflammatory factors including TNF-*α*, interleukin 1*β* (IL-1*β*), and interleukin 6 (IL-6), is the downstream protein of TLR4 [17]. In the central nervous system, TLR4 is mainly expressed in glial cells with very low-level expression in neurons [18]. TLR4 is seen upregulated in the brains of AD patients and AD model mice [19]. The toxic A*β* leads to glial activation and enhances the phagocytosis function of glial cells through TLR4 [20]. Given that the TLR4 alters in AD progression, targeting TLR4 is a promising direction in halting AD. It has been demonstrated that suppressing TLR4 shows a protective effect in AD pathology through an anti-inflammatory mechanism [21, 22].

Trilobatin (TLB), isolated from *Lithocarpuspolystachyus* Rehd., has been shown to alleviate neuroinflammation and oxidative stress in an ischemia/reperfusion brain injury mouse model [23]. In this model, TLB exerts neuroprotective effects through the Nrf2/Kelch-like ECH-associated protein 1 (Keap-1) signaling pathway. A recent study shows that TLB can delay aging through an antioxidative mechanism in *Caenorhabditis elegans* [24]. Moreover, TLB was found to modulate TLR4 function in the ischemia/reperfusion brain injury mouse model [23]. Thus, we hypothesize that TLB can alleviate AD-related neuroinflammation through the TLR4 pathway and attenuate AD pathology. To address this hypothesis, we treated 3×FAD AD model mice with TLB and evaluated the effects of TLB on AD-related pathology and memory function. We found that TLB attenuated memory deficits, alleviated Tau and A*β* pathology, modulated spine plasticity, protected neuronal loss, and inhibited gliosis in the 3×FAD AD mouse model. We think that TLB might alleviate glial activation through the TLR4 pathway. Our results uncovered a natural compound TLB that might halt AD progression through multiple pathways. It might provide a potential therapeutic approach involving TLR4 inhibition in AD treatment.

## 2. Materials and Methods

### 2.1. Animals

C57BL/6J mice (male, 18∼26 g, 6∼8 weeks old) were purchased from the Laboratory Animal Center of Guizhou Medical University (Guizhou, China). 3×FAD transgenic AD mice (APP Swedish, MAPT P301L, and PSEN1 M146V) were purchased from Beijing iBio Logistics Co., Ltd. (Beijing, China). Mice were kept under a 12 h light-dark cycle with full access to food and water. All procedures involving animals were preapproved by the Institutional Animal Care and Use Committee of Guizhou Medical University.

### 2.2. Drug Treatment and Experimental Groups

Four-month-old wild-type (WT) mice and 3×FAD transgenic AD mice were used for all experiments. TLB was dissolved in saline for gavage. The 10 mg/kg or 20 mg/kg dose of TLB (10 mg/kg and 20 mg/kg) was given as reported in earlier studies [23]. Mice were divided into five groups:

*WT:* saline was administered by gavage in place of TLB solution.

*WT+TLB_H_:* WT mice were treated with TLB (high dose) by gavage at a dose of 20 mg/kg once a day for 12 weeks.

*3×FAD:* the 3×FAD transgenic AD mice were treated with saline by gavage in place of TLB.

*3×FAD+TLB_L_:* the 3×FAD transgenic AD mice were treated with TLB (low dose) by gavage at a dose of 10 mg/kg once a day for 12 weeks.

*3×FAD+TLB_H_:* the 3×FAD transgenic AD mice were treated with TLB (high dose) by gavage at a dose of 20 mg/kg once a day for 12 weeks.

After 12 weeks of TLB gavage, the mice were humanely killed with an overdose of sodium pentobarbital (80 mg/kg, i.p.). Then, samples were perfused with 0.9% saline transcardially for 5 min. Mouse brains were removed. One hemisphere of each brain was used for biochemical analysis, and the other hemisphere was fixed in 4% paraformaldehyde for morphological analysis.

### 2.3. Morris Water Maze (MWM) Test

The effect of TLB on the memory performance of mice was analyzed by the MWM test [25]. The maze (110 cm in diameter) was filled with ~20°C opacified water. Before the test, mice were trained to habituate the maze. During training, the mice were allowed to swim for 60 s to find the platform (10 cm in side length) in the second quadrant. The platform was set 1 cm beneath the water surface. The mice were reset at the platform when they failed to find the platform in 60 s. The mice were trained twice a day with a 30 min interval between training sessions. The platform was removed one day after the training. The mice were left in the fourth quadrant and allowed to swim. The swimming trials were recorded using a camera set above the maze. The time spent in the target quadrant (second quadrant) and times of crossing the platform were analyzed as an indicator of memory performance. The test of each mouse was repeated 3 times, and the average time of these three trials was recorded.

### 2.4. Congo Red Staining

Congo red staining was conducted using a Congo red amyloid stain kit (G1532, Solarbio Life Science, Beijing, China). Paraffin-embedded brains were sectioned using a microtome (RM2235, Leica, Germany), immersed in Congo red solution for 25 min, and then washed with 0.1 M PBS. After being stained with hematoxylin for 30 sec, the sections were dehydrated in gradient alcohol. Pictures were acquired using a microscope (CX23, Olympus, Japan). Six mice per group were included in the experiment. Three separate serial sections per mouse were processed for Congo red staining analysis.

### 2.5. Immunohistochemical (IHC) Staining

Brain tissues were sectioned using a microtome (RM2235, Leica, Germany). After antigen recovery using sodium citrate, the sections were incubated with primary antibodies mouse anti-GFAP (3670s, 1 : 200, Cell Signaling Technology, USA), A*β* (ab201060, 1 : 200, Abcam, USA), phosphor-Tau (ab32057, 1 : 150, Abcam, USA), and rabbit anti-NeuN (ab128886, 1 : 200, Abcam, USA). The sections were developed with 3,3′-diaminobenzidine (DAB) kits (Cat#CW2069, CWBio, China) according to the manufacturer's protocol. Images were obtained using a microscope (CX23, Olympus, Japan). Six mice per group were included in the experiment. Three separate serial sections per mouse were processed for immunostaining analysis.

For immunofluorescence labeling, frozen sections (25 *μ*m) were cut using a freezing microtome (CM1950, Leica, Wetzlar, Germany). After blocking with 5% BSA containing 0.5% Triton X-100 for 40 min, the floating sections were incubated with primary antibodies A*β* (ab201060, 1 : 200, Abcam, USA) and Iba1 (ab178846, 1 : 200, Abcam, USA). After being rinsed in water, the sections were incubated in the secondary antibody Alexa Fluor 488 (A-21206, Thermo Fisher Scientific, MA, USA). Nuclei were labeled using DAPI (H-1020, Vector Labs, CA, USA). Images were acquired using a confocal microscope (LSM 900, Carl Zeiss, Germany). Six mice per group were included in the experiment. Three separate serial sections per mouse were processed for immunostaining analysis.

### 2.6. Nissl Staining

The Nissl staining was conducted using a Nissl staining kit (G1434, Solarbio Life Science, Beijing, China) according to the manufacturer's protocol. Paraffin-embedded brain tissues were sectioned using a microtome (RM2235, Leica, Germany). Then, the sections were dewaxed and rehydrated before being immersed into methylene blue stain solution for 10 min. After being immersed into the Nissl differentiation solution for 3 sec, the sections were rinsed in water. At last, the sections were dehydrated in pure alcohol. Pictures were acquired using a microscope (CX23, Olympus, Japan). Six mice per group were included in the experiment. Three separate serial sections per mouse were processed for Nissl staining analysis.

### 2.7. Western Blot

Hippocampal tissue was homogenized in the protein extraction buffer containing protease and phosphatase inhibitors. After centrifugation, the protein supernatant was collected. Protein concentration was measured with a Protein Quantitative Analysis Kit (Biocolors, Shanghai, China). The supernatant was then mixed with the loading buffer and boiled at 99°C for 10 min. The samples were separated by SDS-PAGE and transferred to PVDF membranes (Millipore, Billerica, MA, USA). The membranes were blocked in 5% nonfat milk at room temperature for 1 h, then incubated overnight at 4°C with the following primary antibodies: A*β* (ab217153, 1 : 1000, Abcam, USA), BACE1 (ab108394, 1 : 1000, Abcam, USA), sAPP*β* (ab32136, 1 : 1000, Abcam, USA), p-GSK3*β* Y216 (ab68476, 1 : 1000, Abcam, USA), GSK3*β* (ab93926, 1 : 1000, Abcam, USA), p-Ser396 Tau (9632S, 1 : 1000, Cell Signaling Technology, USA), p-Ser202 Tau (39357S, 1 : 1000, Cell Signaling Technology, USA), Tau (ab80579, 1 : 1000, Abcam, USA), PSD95 (ab2723, 1 : 1000, Abcam, USA), SNAP25 (ab109105, 1 : 1000, Abcam, USA), Syn1 (ab254349, 1 : 1000, Abcam, USA), SYP (ab32127, 1 : 1000, Abcam, USA), VAMP1 (ab151712, 1 : 1000, Abcam, USA), TLR4 (ab13556, 1 : 1000, Abcam, USA), MYD88 (ab219413, 1 : 1000, Abcam, USA), TRAF6 (ab33915, 1 : 1000, Abcam, USA), p-NF*κ*B (ab76302, 1 : 1000, Abcam, USA), NF*κ*B (ab32536, 1 : 1000, Abcam, USA), TNF-*α* (ab66579, 1 : 1000, Abcam, USA), IL-1*β* (ab200478, 1 : 1000, Abcam, USA), IL-6 (ab208113, 1 : 1000, Abcam, USA), and *β*-actin (ab8226, 1 : 1000, Abcam, USA). After incubating with the secondary antibody at room temperature for 1 h, the membranes were interacted with electrochemiluminescence reagents (Bio-Rad, Hercules, CA, USA) to visualize the immunoblot signals. ImageJ software was used to measure band densities, and protein expression levels were normalized to *β*-actin intensity.

### 2.8. Dendritic Spine Analysis

Brain tissues were fixed in 4% PFA for 4 hrs. Sections of 250 *μ*m were made using a vibratome (VT1200S, Leica, Germany) and were mounted on glass slides. Lucifer yellow fluorescent dye (4% in lithium chloride, L453, Thermo Fisher Scientific) was loaded into a pipette and injected into the neurons in the hippocampal area. Briefly, the dye was injected into a neuron with a ~2 nA current for 20 min until the whole dendritic branches were visualized under a fluorescent microscope. The 3D *z*-stack dendritic spine images were obtained using a confocal microscope (LSM 880, Carl Zeiss, Germany). The number of dendritic spines was analyzed using Imaris software (Oxford instruments). Six mice per group were included in the experiment. Three separate serial sections per mouse were processed for dendritic spine analysis.

### 2.9. Statistical Analysis

Results were collected from three independent experiments. The data were presented as mean ± SD and were analyzed using SPSS 25.0 (IBM Corporation, Armonk, NY, USA) and GraphPad Prism 7.0 (GraphPad Software, Inc., CA, USA). The differences were assessed by one-way analysis of variance. *p* < 0.05 was defined as statistically significant.

## 3. Results

### 3.1. TLB Ameliorated Cognitive Deficits in 3×FAD Mice

**Figure 1:**
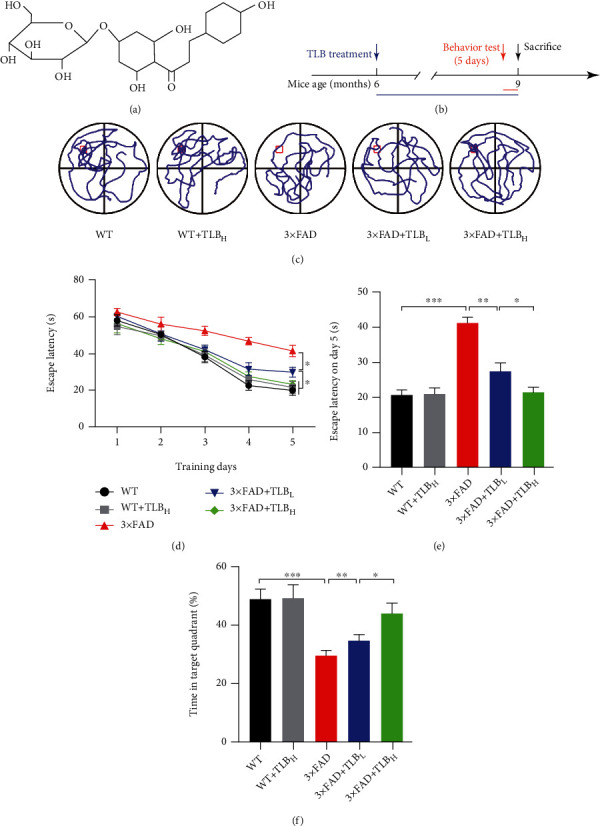
TLB ameliorated cognitive deficits in 3×FAD mice. (a) Chemical structure of TLB (C_21_H_24_O_10_, molecular weight = 436.4). (b) Experimental design. (c) Representative paths in the MWM test. (d) The escape latency of each mouse group tested from day 1 to day 5. (e) The escape latency on day 5. (f) Time spent in the target quadrant in the MWM test. *n* = 6 per group. ^∗^*p* < 0.05; ^∗∗^*p* < 0.01; ^∗∗∗^*p* < 0.001.

The MWM test was conducted to determine whether the cognitive impairments of 3×FAD AD mice were rescued by TLB. The MWM test showed that the escape latency was progressively decreased from day 1 to day 5 (Figure 1(d)). However, the 3×FAD mice showed an elevated escape latency compared to WT mice on day 5. In contrast, 3×FAD AD mice treated with a low level of TLB (10 mg/kg) showed a decreased escape latency compared to 3×FAD mice (Figure 1(e)). Moreover, the 3×FAD mice treated with a high dose of TLB (20 mg/kg) showed a significantly lower escape latency compared to 3×FAD mice treated with a low dose of TLB (Figure 1(e)). It is noteworthy that the escape latency in 3×FAD mice treated with a high dose of TLB has no differences compared to that in WT mice. Furthermore, we compared the time spent in the target quadrant from each mouse group. The 3×FAD mice showed a shorter time in the target quadrant than WT mice. And the 3×FAD mice treated with low-dose TLB showed a longer swimming time in the target quadrant compared to 3×FAD mice. The 3×FAD mice treated with high-dose TLB showed a longer swimming time in the target quadrant compared to low-dose TLB-treated mice (Figure 1(f)).

### 3.2. TLB Alleviated A*β* Deposition in 3×FAD Mice

**Figure 2:**
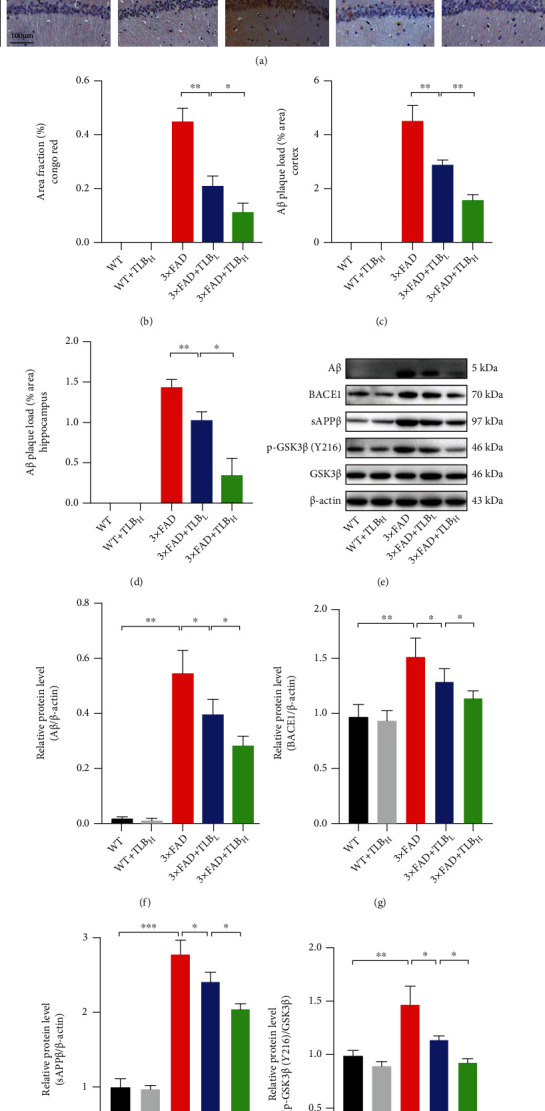
TLB alleviated A*β* deposition in 3×FAD mice. (a) Congo red staining and A*β* IHC staining in the hippocampal area and cortex. (b) Comparison of Congo red staining analysis in the hippocampal area. (c) Comparison of A*β*-positive plaque in the cortex. (d) A*β* plaque load in the hippocampal CA1 area. (e–i) Western blot and quantitative analysis of A*β*, BACE1, sAPP*β*, p-GSK3*β*, and GSK3*β* in hippocampal tissues. *n* = 6 per group. ^∗^*p* < 0.05; ^∗∗^*p* < 0.01; ^∗∗∗^*p* < 0.001.

To investigate the effect of TLB on A*β* deposition, the Congo red staining and A*β* IHC staining were performed. Both revealed that the A*β* plaque load is significantly increased in 3×FAD AD mice. Compared to the 3×FAD mice, the mice treated with a low dose of TLB showed a significant decrease of the A*β* plaque load in the cortex and hippocampus. Furthermore, a high dose of TLB showed a remarkable A*β* load alleviation effect than a low dose of TLB in 3×FAD mice (Figures 2(a)–2(d)). Next, we examined the effect of TLB on amyloidogenic processing in 3×FAD mice. We found that the BACE1 and sAPP*β* protein levels were increased in 3×FAD mice compared to WT mice. And both a low dose and a high dose of TLB decreased the BACE1 and sAPP*β* levels in 3×FAD mice. Furthermore, TLB reversed the phosphorylation of GSK3*β* levels in 3×FAD mice (Figures 2(e)–2(i)).

### 3.3. TLB Inhibited Tau Phosphorylation in the AD Mouse Model

**Figure 3:**
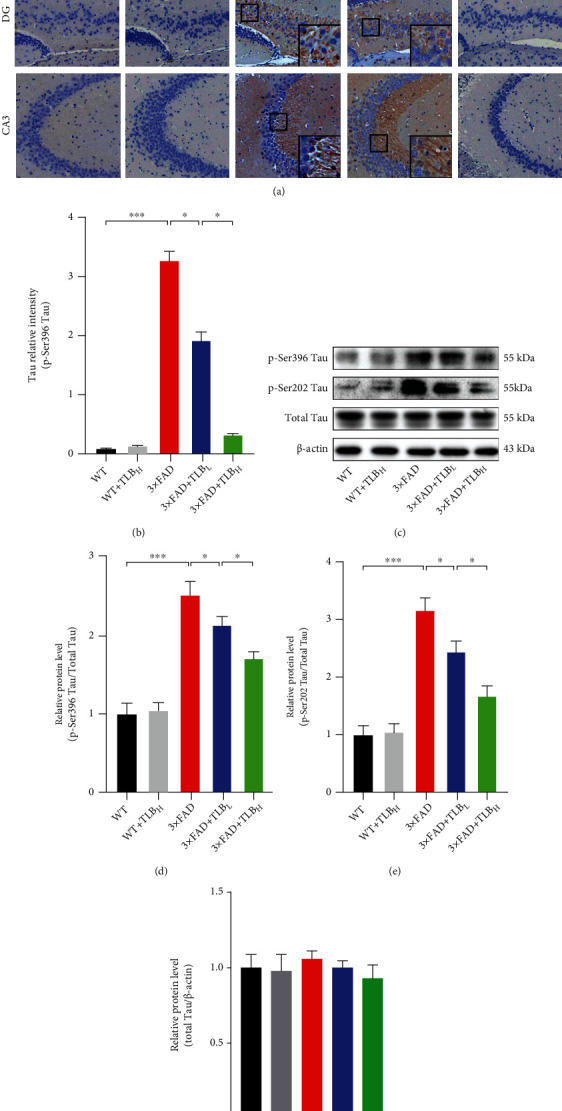
TLB inhibited Tau phosphorylation in the AD mouse model. (a) Representative images of p-Ser396 Tau IHC staining. (b) Relative intensity of p-Ser396 Tau in hippocampal subregions. (c–f) Western blot and quantification for phosphorylated Tau at Ser396 and Ser202 sites and total Tau in the hippocampus. *n* = 6 per group. ^∗^*p* < 0.05; ^∗∗^*p* < 0.01; ^∗∗∗^*p* < 0.001.

The Tau pathology was assessed in the 3×FAD AD mouse model. Tau IHC staining showed that p-Ser396 Tau pathology was remarkable in 3×FAD mice. The intensity of p-Ser396 Tau was significantly lower in hippocampal subregions of low-dose TLB-treated AD model mice than in those of WT mice. And a high dose of TLB showed a much stronger Tau pathology alleviation effect than a low dose of TLB (Figures 3(a) and 3(b)). Western blots further revealed that Tau phosphorylation at Ser396 and Ser202 sites was remarkably decreased in 3×FAD+TLB_L_ and 3×FAD+TLB_H_ mice compared to 3×FAD mice (Figures 3(c)–3(f)).

### 3.4. TLB Reduced Neuronal Loss and Synaptic Degeneration in 3×FAD Mice

**Figure 4:**
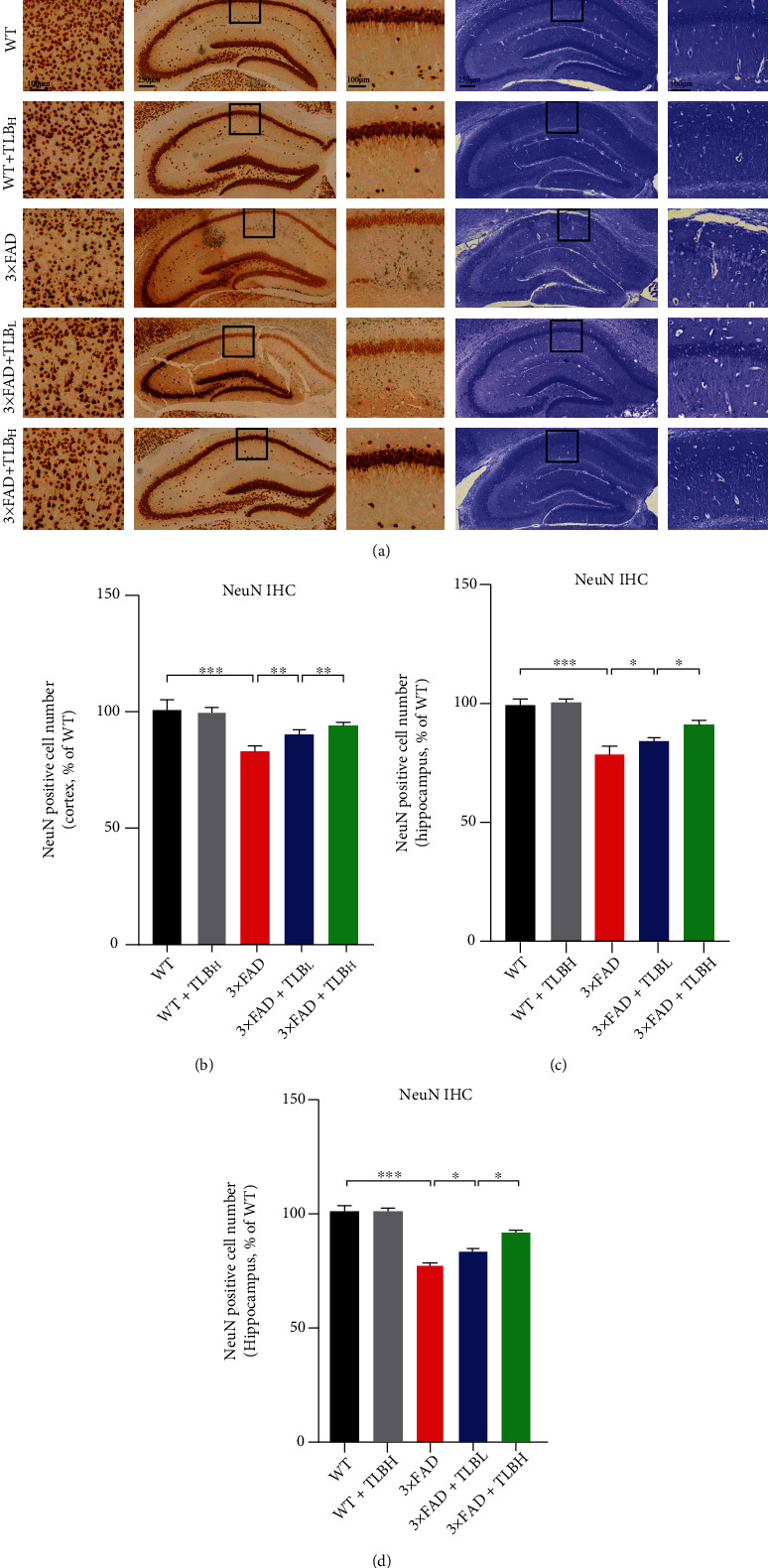
TLB reduced neuronal loss in 3×FAD mice. (a) Representative figures of NeuN staining in the cortex and hippocampus and Nissl staining in hippocampal regions. (b, c) Quantification of neuronal content in the cortex and hippocampus from NeuN IHC staining results. (d) Quantitative analysis of the surviving neuron number in the hippocampus from Nissl staining results. *n* = 6 per group. ^∗^*p* < 0.05; ^∗∗^*p* < 0.01; ^∗∗∗^*p* < 0.001.

In order to assess whether oral administration of TLB affects the neuronal number in 3×FAD mice, we performed neuronal marker NeuN IHC staining and Nissl staining in the cortex and hippocampus. We found a remarkable reduction of the NeuN-positive cell number in the cortex and hippocampus of 3×FAD mice compared to WT mice. Consistently, Nissl staining showed that the Nissl-positive cell number was suppressed in 3×FAD mice compared to WT mice. In contrast, treatment with low and high doses of TLB clearly increased the NeuN- and Nissl-positive cell number in the AD mouse model. Note that the neuronal number in the 3×FAD+TLB_H_ group was higher than that in the 3×FAD+TLB_L_ group (Figure 4).

**Figure 5:**
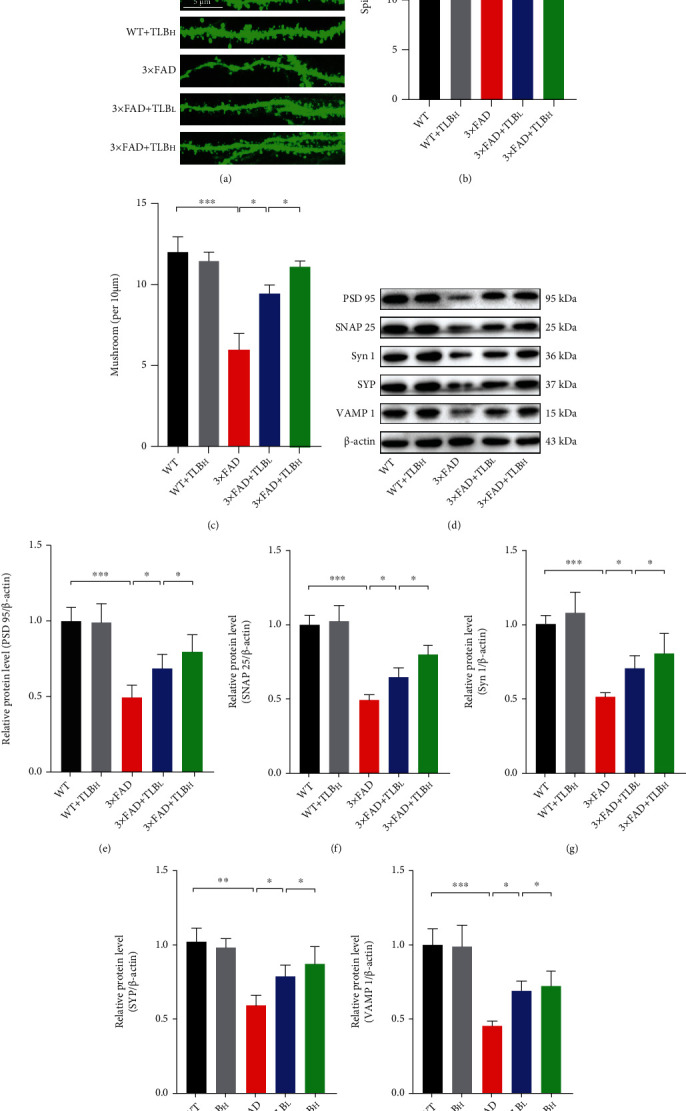
TLB alleviated synaptic degeneration in AD model mice. (a) Representative images of spine morphology in each group. (b, c) Quantitative analysis of total spine density and mushroom-type spine density. (d) The representative blots of PSD95, SNAP25, Syn1, SYP, and VAMP1 in each group. (e–i) Quantitative analysis of PSD95, SNAP25, Syn1, SYP, and VAMP1 protein levels. *n* = 6 per group. ^∗^*p* < 0.05; ^∗∗^*p* < 0.01; ^∗∗∗^*p* < 0.001.

To further test the effect of TLB on synaptic density, we conducted spine morphology analysis. The total spine number and mushroom spine number were suppressed in 3×FAD AD model mice compared to WT mice. However, low and high doses of TLB oral treatment reduced the total and mushroom spine density loss in hippocampal areas of AD model mice (Figures 5(a)–5(c)). Western blot further revealed that the synapse-associated protein including PSD95, SNAP25, Syn1, SYP, and VAMP1 expression was suppressed in 3×FAD AD model mice. In contrast, low and high doses of TLB treatment reversed the synapse-associated protein loss in 3×FAD AD model mice (Figures 5(d)–5(i)).

### 3.5. TLB Alleviated Glial Activation in AD Model Mice

**Figure 6:**
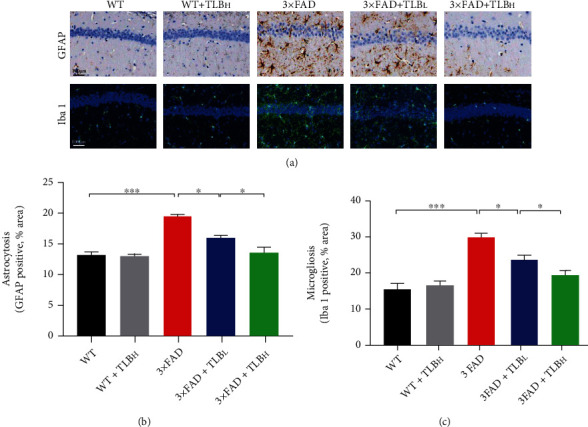
TLB alleviated glial activation in AD model mice. (a) Representative images of GFAP and Iba1 staining in the hippocampal area. (b) Quantification of GFAP staining in each group. (c) Quantification of Iba1 staining in each group. *n* = 6 per group. ^∗^*p* < 0.05; ^∗∗^*p* < 0.01; ^∗∗∗^*p* < 0.001.

In order to test the effect of TLB on glial activation, hippocampal sections were immunostained with antibodies including astrocyte marker GFAP and microglial marker Iba1. A significant increase of the GFAP- and Iba1-positive cell number was detected in the 3×FAD mouse brains compared with WT mice. And a remarkable reduction of the GFAP- and Iba1-positive cell number was detected in the hippocampal CA1 area of the 3×FAD mice compared with the vehicle-treated mice. Furthermore, a significant difference of the GFAP- and Iba1-positive cell number was noticed between the low- and high-dose TLB-treated 3×FAD AD mice (Figure 6).

### 3.6. TLB Ameliorated Neuroinflammation through Reducing TLR4 in AD Model Mice

To further study the potential mechanism underlying the effect of TLB on AD pathology-related neuroinflammation, we tested the TLR4 signaling pathway proteins. We found that TLR4, MYD88, TRAF6, p-NF*κ*B/NF*κ*B, TNF-*α*, IL-1*β*, and IL-6 were elevated in 3×FAD mice compared to WT mice. And TLB treatment induced a reduction of TLR4, MYD88, TRAF6, p-NF*κ*B/NF*κ*B, TNF-*α*, IL-1*β*, and IL-6 levels compared to the vehicle-treated 3×FAD mice. Furthermore, a significant difference was observed between the low and high-dose TLB-treated mice.

## 4. Discussion

In the present study, we identified that TLB might alleviate A*β* deposition, Tau pathology, synaptic degeneration, glial activation, and memory impairment in the AD mouse model. And the TLR4-MYD88-NK*κ*B pathway might be involved in the anti-neuroinflammatory effect in the process. We show for the first time that TLB reverses AD pathology and memory impairment in 3×FAD mice, indicating a promising drug candidate for halting AD progression.

In a recent study, Chen et al. [26] showed that TLB protects HT22 cell death induced by A*β*_25-35_ through the ROS/p38/caspase 3 pathway. However, the study has not shown the effect of TLB in the AD *in vivo* model. In the present study, we showed that TLB has reversed the memory impairment phenotype in the 3×FAD AD mouse model using the MWM test. Notably, the high dose of TLB improved the memory function of 3×FAD mice to the level of WT mice (Figures 1(c)–1(f)).

Since AD is a neurodegenerative disease characterized by A*β* deposition and p-Tau aggregation, we therefore tested the effect of TLB on A*β* and p-Tau pathology. Specifically, we applied Congo red and A*β* IHC staining in hippocampal and cortical regions. We found that TLB dramatically reduced the A*β* burden (Figures 2(a)–2(d)). More importantly, we observed that TLB inhibited the expression of BACE1 and sAPP*β*, suggesting that TLB can break the loop of the BACE1-mediated amyloidogenesis. And TLB also suppressed the hyperphosphorylation of GSK3*β*, which acts as a Tau kinase (Figures 2(e)–2(i)). As we know, GSK3*β* modulates BACE1 expression, and A*β* can drive the BACE1 level to increase. Thus, it is likely that the reduced amyloid deposition by TLB might be through breaking the vicious cycle of the A*β* burden, A*β*-induced GSK3*β* activation, and GSK3*β*-induced BACE1 expression. Moreover, TLB inhibited the Tau hyperphosphorylation at Ser396 and Ser202 sites, as evidenced by western blot and IHC staining (Figures 3). TLB may also suppress the activation of GSK3*β*, which is characterized by GSK3*β* hyperphosphorylation. This finding suggests that TLB ameliorates Tau hyperphosphorylation by inhibiting GSK3*β* activation.

The neuronal loss in the hippocampal region accounts for memory deficits in AD [27]. We examined the effect of TLB on neuronal density in the hippocampus and cortex. We found that a low dose and high dose of TLB treatment rescued the neuronal loss in 3×FAD AD mice, as examined by NeuN IHC and Nissl staining methods (Figure 4). This neuroprotective effect of TLB is consistent with the finding in an earlier study of oxygen deprivation and reoxygenation on primary cortical neurons [23].

Given that synaptic degeneration is also involved in the process of AD-related memory impairment [28], we then tested the effect of TLB on synaptic pathological plasticity. Spine density analysis unexpectedly revealed synaptic pathology in the 3×FAD AD mouse hippocampal area (Figures 5(a)–5(c)). Our finding of rescued total spine density and mushroom spine density in TLB-treated 3×FAD mice showed a synaptic protective effect of TLB. Additionally, western blot analysis revealed that the loss of synaptic proteins in 3×FAD mice was attenuated by orally treated TLB (Figures 5(d)–5(i)). It is likely that the memory improvement by TLB was due to the spine density increase and synaptic protein upregulation. In line with this, preserving dendritic spine density leads to cognitive improvement and memory loss attenuation in the 5×FAD AD mouse model [29].

Extracellular A*β* deposition and intracellular p-Tau aggregation might trigger reactive gliosis which leads to inflammatory factors [30]. And reactive gliosis has been proved to drive the pathogenic cascades of AD [31]. Previous studies of anti-inflammatory strategies showed improvements in neurodegenerative disease models [32]. Thus, we applied GFAP and Iba1 immunostaining analysis to observe the number of astrocytes and microglia. Increased astrocytosis and microgliosis were observed in 3×FAD mouse brains, whereas reduced gliosis was observed in the TLB-treated AD model brain samples, suggesting a strong anti-inflammatory effect of TLB (Figure 6). The data from Gao et al. [23] are consistent with ours in that TLB exerts an inactivation effect on glial cells. Studies have shown that, in AD, glial cells' phagocytosis function is impaired, which leads to the toxic A*β* burden [33]. Thus, we reckoned that the glial phagocytosis function is enhanced after TLB treatment, and the A*β* burden is reduced finally.

**Figure 7:**
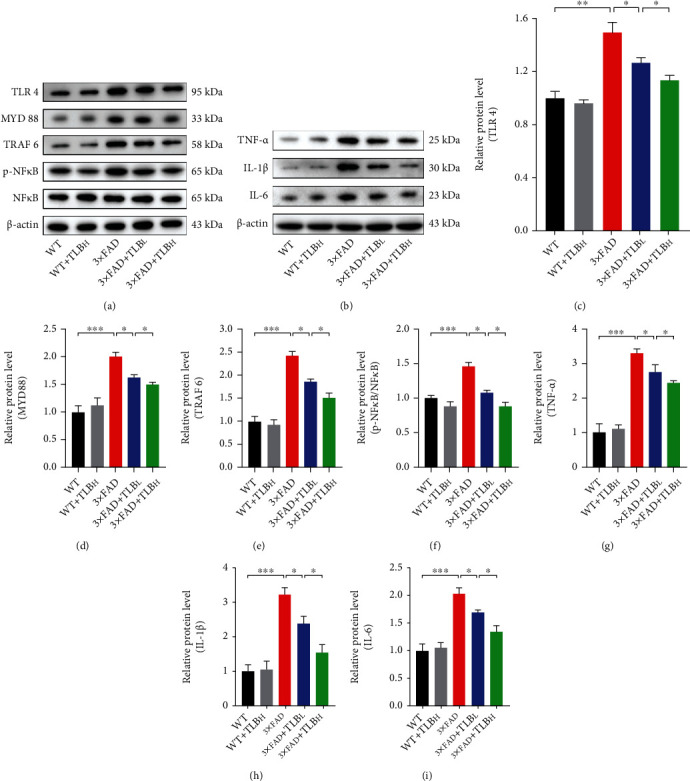
TLB ameliorated neuroinflammation through reducing TLR4 in AD model mice. (a) Representative western blots of TLR4, MYD88, TRAF6, p-NF*κ*B, and NF*κ*B. (b) Representative western blots of TNF-*α*, IL-1*β*, and IL-6. (c–f) Quantification of TLR4, MYD88, TRAF6, and p-NF*κ*B/NF*κ*B levels in each group. (g–i) Quantification of TNF-*α*, IL-1*β*, and IL-6 levels in each group. *n* = 6 per group. ^∗^*p* < 0.05; ^∗∗^*p* < 0.01; ^∗∗∗^*p* < 0.001.

Although we found that TLB can inhibit reactive gliosis in AD mouse brains, the molecular mechanisms responsible for this inhibition remained unknown. Earlier studies revealed that the anti-inflammatory effect of TLB correlates with inhibiting NF*κ*B phosphorylation *in vivo* and *in vitro* [34]. Based on this notion, we detected the TLR4-MYD88-NF*κ*B pathway proteins. As expected, we found that, in 3×FAD mouse brains, TLR4-MYD88 pathway proteins were overexpressed, accompanied by NF*κ*B hyperphosphorylation. TLB reversed the changes in this AD mouse model, suggesting that the anti-inflammatory effect might be TLR4-MYD88-NF*κ*B pathway-dependent (Figure 7). Moreover, we demonstrated that TLB reduces the inflammatory factors which are regulated by NF*κ*B phosphorylation. In line with this, studies have found that the loss of function or inhibition of TLR4 suppresses AD progression in the mouse model [35, 36]. Moreover, previous studies showed that inhibiting the TLR4 pathway alleviated motor impairment and dopaminergic neuron death in the Parkinson's disease mouse model [37]. And Kwilasz et al. showed that TLR4 antagonists prevented the production of proinflammatory factors and motor dysfunction in the experimental autoimmune encephalomyelitis mouse model [38]. All these studies have shown that TLR4 might be a promising target in neuroinflammation treatment. Given the fact that inflammatory factors contribute to neuronal loss, synaptic loss, and behavior impairments in AD progression, we think that the TLR4-MYD88-NF*κ*B-dependent anti-inflammatory effect of TLB might be accountable for its neuroprotective effect.

Due to the complicated pathophysiological changes in AD progression, drugs targeting A*β* failed in halting AD-related memory deficits and pathological changes. We think that targeting multiple pathways for AD intervention, such as anti-inflammation, A*β* reduction, and Tau hyperphosphorylation inhibition, might be an effective treatment plan. According to the experiments above, we have a reason to consider that TLB seems to have multiple targets on halting AD progression.

## 5. Conclusion

In summary, we uncovered TLB for AD therapy by exploring multiple pathway mechanisms including A*β* burden reduction, Tau hyperphosphorylation inhibition, and anti-inflammation. TLB was effective in reducing neuronal loss, alleviating synaptic degeneration, and ameliorating memory deficits in the AD mouse model. More studies and further clinical trials to test its efficacy would be necessary.

## Data Availability

All processed data and models used during the study are available from the corresponding authors by request. But the raw data required to reproduce these findings cannot be shared at this time as the data also forms part of an ongoing study.
